# Platelet-to-lymphocyte ratio, a novel biomarker to predict the severity of COVID-19 patients: A systematic review and meta-analysis

**DOI:** 10.1177/1751143720969587

**Published:** 2022-02

**Authors:** Daniel Martin Simadibrata, Bashar Adi Wahyu Pandhita, Muammar Emir Ananta, Tamara Tango

**Affiliations:** Faculty of Medicine, Universitas Indonesia, Jakarta, Indonesia

**Keywords:** Platelet-to-lymphocyte ratio, severity, COVID-19, coronavirus, SARS-CoV-2

## Abstract

Platelet-to-lymphocyte ratio (PLR), a novel inflammatory marker, has been suggested to predict the severity of COVID-19 patients. This systematic review aims to evaluate the association between PLR levels on admission and the severity of COVID-19 patients. A systematic literature search was done on 23 July 2020 to identify peer-reviewed studies, preprints, and grey literatures. Research articles comparing the PLR value on admission in adult patients with COVID-19 with varying degrees of severity were included in the analysis. The following keywords were used for the search: “COVID-19”, “PLR”, “severity”, and “mortality”. A total of seven studies were included in the meta-analysis, six of which were conducted in China. From a total of 998 participants included, 316 (31.7%) had severe diseases; and those in the severe group were generally older and had underlying diseases compared to the non-severe group. In comparison to non-severe patients, the meta-analysis showed that severe COVID-19 patients had higher PLR levels on admission (SMD 0.68; 95%CI 0.43-0.93; I^2^ =58%). High PLR levels on admission were associated with severe COVID-19 cases. Therefore, the on-admission PLR level is a novel, cost-effective, and readily available biomarker with a promising prognostic role for determining the severity of COVID-19 patients.

## Introduction

 Coronavirus Disease 2019 (COVID-19) is a disease caused by the severe acute respiratory syndrome coronavirus-2 (SARS-CoV-2), a virus thought to start as a zoonotic infection in Wuhan in late December 2019.^1^ The disease was declared by the World Health Organization (WHO) as a pandemic on 11 March 2020 and has infected more than 100 countries worldwide. As of 26 July 2020, a total number of 15 785 641 cases and 640 016 deaths attributed to COVID-19 were recorded, only months after its emergence.^2^

COVID-19 is known for being infectious and simultaneously manifesting in different organs aside from the pulmonary system.^3–5^ Patients infected with COVID-19 present a wide range of clinical conditions – ranging from asymptomatic infections, minimal symptoms to fatal respiratory distress. Although the majority of COVID-19 cases were classified as mild, involving flu-like symptoms to mild pneumonia, up to 20% of mild/moderate cases progressed to acute respiratory distress syndrome (ARDS).^6^ Additionally, patients with relatively normal clinical conditions can rapidly deteriorate and worsen within a few days, making clinical presentation an unreliable prognostic predictor of COVID-19. Thus, a more objective indicator is required to accurately assess and stratify the prognosis of COVID-19 patients upon admission to healthcare services.

Immunological studies have shown that high levels of proinflammatory cytokines, known as a cytokine storm, are the hallmark characteristic of severe COVID-19 cases. This extreme elevation of cytokines causes a massive proinflammatory response resulting in Multiple Organ Dysfunction Syndrome (MODS) and ARDS, which subsequently leads to mortality in COVID-19 patients.^7^ Therefore, in theory, inflammatory markers can be used to assess the severity and mortality risk of COVID-19 patients.

Platelet-to-lymphocyte ratio (PLR) is a novel marker of inflammation, which is inexpensive and readily available in clinical settings. PLR has been used in various diseases, such as cardiovascular diseases and autoimmune diseases, as a predictor of inflammation and mortality.^8,9^ Due to the rapid involvement of inflammatory processes in COVID-19, severe COVID-19 patients have demonstrated elevated PLR levels on admission.^10,11^ This suggests the potential use of this inflammatory marker to determine the prognosis of COVID-19 patients, especially in resource-limited settings. Therefore, this systematic review aims to review the prognostic value of PLR levels on admission to determine the severity and mortality of COVID-19 patients.

## Methods

### Protocol and registration

This systematic review was written in compliance with the Preferred Reporting Items for Systematic Reviews and Meta-analyses (PRISMA) Checklist (Table S1). Prior to the writing of this systematic review, a protocol was formulated and registered in the International Prospective Register of Systematic Reviews (PROSPERO) on 2 June 2020 (CRD42020189369).

### Eligibility criteria

We included cohort studies evaluating the difference in PLR levels on admission in adults (>18 years old) with confirmed COVID-19 (diagnosed using RT-PCR) categorized based on disease severity (severe and non-severe patients), and/or mortality (survivor and non-survivor). Case series, correspondences, review articles, non-research articles, and letters to the editor were excluded from the study. We only included papers written and published in English. Otherwise, no other exclusion criteria were applied. The severity of the disease was defined by the WHO-China Joint Mission on COVID-19 report.^12^ Severe COVID-19 was defined as patients that met any of the following criteria: respiratory frequency ≥30x/minute, blood oxygen saturation ≤93%, PaO2/FiO2 ratio <300, and/or lung infiltrates >50% of the lung field within 24-48 hours.

### Search strategy

The keywords used in the search strategy were derived from the following key concepts “COVID-19”, “platelet-to-lymphocyte”, “severity”, and “mortality”; and were adapted to the respective databases (Table S2). A systematic literature search was finalized on 23 July 2020 to identify peer-reviewed papers published in four databases (Ovid MEDLINE, EMBASE, SCOPUS, and the Cochrane Library). Additionally, manual handsearching was done for preprints in two databases (MedRxiv and SSRN), and for grey literatures in two databases (WHO COVID-19 Global Research Database and Center for Disease Control and Prevention COVID-19 Research Article). We also performed forward and backward tracing of references from relevant articles to identify additional papers missed from database searching.

### Study selection

All articles retrieved from the searches were exported to EndNote X9 reference manager. After de-duplication of articles, publications were screened based on its titles and abstracts, and the remaining publications were screened according to the full text. This study selection process was carried out by two independent reviewers (DMS and BAW). Any disagreement regarding the study selection was resolved by the inclusion of a third party.

### Quality assessment and data extraction

All included articles’ data were extracted by two independent reviewers (DMS and BAW). The risk of bias assessment was done using the Newcastle Ottawa Scale (NOS). A standardized data extraction form was used to gather relevant data from the selected articles. Data extracted included: first author and publication year, publication type, country of study, study design, baseline characteristics of patients (age, gender, and underlying diseases), and the outcome of the study (PLR values).

### Statistical analysis

For the quantitative analysis, we exported quantitative data from all eligible studies to Review Manager software 5.3 (Cochrane Collaboration) and performed a meta-analysis. We extrapolated the mean and standard deviation of studies with non-normal data using the available median and interquartile range (IQR), according to Hozo *et al.*^13^ The inverse variance method was used to obtain the effect estimate in the form of a standardized mean difference (SMD) and the 95% Confidence Interval (CI). The statistical heterogeneity between the studies was assessed using Cochrane chi-square and I^2^. If there was significant heterogeneity (I^2^ > 50%), the random-effects model was used to calculate the pooled effect size; otherwise, the fixed-effects model was used. Funnel plot analysis was used to qualitatively evaluate the risk of publication bias by comparing the SMD with the standard error of the natural log of SMD. We performed a sensitivity analysis by omitting one study at a time to identify the source of heterogeneity. All *p*-values were two-tailed and statistical significance was considered if *p* < 0.05.

## Results

### Search selection and study characteristics

**Figure 1.**
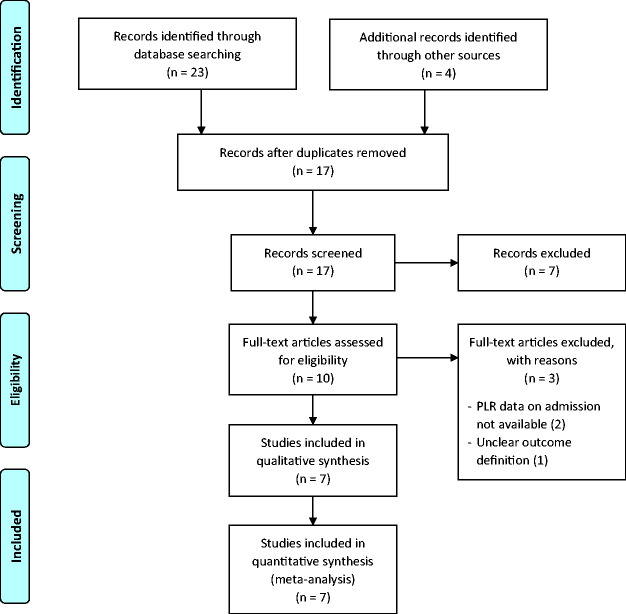
Prisma diagram for study selection in the meta-analysis. A systematic search of the literature was performed from database conception to 23 July 2020.

The literature search from electronic databases resulted in 27 publications, four of which were identified through manual handsearching of relevant literature (Figure 1). Following de-duplication, 17 articles were screened for its titles and abstracts. The remaining ten articles were reviewed for its full-text, and three articles were excluded, leaving a total of seven peer-reviewed articles included in this systematic review.

From a total of seven selected studies, all were peer-reviewed and were retrospective observational studies. Overall, six studies were done in China^10,11,14–17^ and only one was done outside of China, in Turkey.^18^ All studies compared the on-admission PLR values between severe and non-severe COVID-19 patients, except for one that compared the PLR values between severe and moderate COVID-19 patients (Table 1).^17^ Unfortunately, no study explored the prognostic value of PLR on admission to predict mortality. The quality of the included studies is shown in Table S3. Four studies had seven NOS stars, and the other three had six NOS stars. In brief, all studies were of acceptable quality and eligible for inclusion in the meta-analysis.

**Table 1.** Summary of baseline characteristics and study findings of all included studies in the meta-analysis.

No.	Author	Study design	Groups	Sample (N)	Male/female (% of male)	Age (years) Mean ± SD/Median (IQR)	*p*	HT N (%)	*p*	DM N (%)	*p*	CVDN (%)	*p*	PLR value Mean ± SD/Median (IQR)	*p*
1	Qu R	Retrospective Observational	**Non-severe**	27	16/14 (53)	49.4 ± 14.9	0.041*	NR	NR	NR	NR	NR	NR	242.75 ± 173.74	0.414
			**Severe**	3		60.0 ± 5.3		NR		NR		NR		160.02 ± 51.99	
2	Yang AP	Retrospective Observational	**Non-severe**	69	38/31 (55)	42.1 ± 18.6	0.034*	7 (10)	<0.01*	8 (12)	<0.01*	4 (6)	<0.01*	176.7 ± 84.2	<0.001*
			**Severe**	24	18/6 (75)	57.9 ± 11.8		16 (67)		13 (54)		9 (38)		436.5 ± 329.2	
3	Gong J	Retrospective Observational	**Non-severe**	161	72/89 (45)	45.0 (33.0–62.0)	<0.01*	NR	NR	NR	NR	NR	NR	131.0 (96.6–177.4)	0.05
			**Severe**	28	16/12 (57)	63.5 (54.5–72.0)		NR		NR		NR		174.8 (117.7–210.0)	
4	Zhu Z	Retrospective Observational	**Non-severe**	111	73/38 (66)	49.9 ± 15.5	0.03*	23 (21)	0.025*	10 (9)	0.451	4 (4)	0.348	160.00 (129.64–215.00)	0.299
			**Severe**	16	9/7 (56)	57.5 ± 11.7		8 (50)		0 (0)		2 (13)		210.46 (116.33–300.88)	
5	Sun S	Retrospective Observational	**Common**	89	42/47 (47)	47.0 (37.0–54.5)	<0.001*	NR	NR	NR	NR	NR	NR	160.7 (116.7–207.5)	<0.001*
			**Severe**	27	18/9 (67)	62.0 (53.0–71.0)		NR		NR		NR		246.0 (167.9–456.7)	
6	Zhou Y	Retrospective Observational	**Moderate**	140	55/85 (39)	55.9 ± 14.4	NR	NR	NR	NR	NR	NR	NR	181 ± 97	NR
			**Severe**	123	58/65 (47)	63.8 ± 13.9		NR		NR		NR		262 ± 196	
			**Critically severe**	41	25/16 (61)	65.2 ± 12.7		NR		NR		NR			
7	Ok F	Retrospective Observational	**Non-severe**	85	38/47 (45)	47.2 ± 15.7	<0.001*	9 (11)	<0.001*	12 (14)	0.218	2 (2)	<0.001*	143.6 (94.2)	0.072
			**Severe**	54	24/30 (44)	68.3 ± 14.9		24 (44)		12 (22)		17 (32)		197.8 (178.2)	

CVD = Cardiovascular Disease; DM = Diabetes Mellitus; HT = Hypertension; IQR = Interquartile Range; NR = Not reported; PLR = Platelet-to-lymphocyte ratio; *p* = *p*-value; SD = Standard Deviation.

*Statistically significant (*p* < 0.05).

### Baseline characteristics and study findings

A total of 998 participants were included from all the studies, 316 (31.7%) of which had severe disease. Although four studies did not report the baseline comorbidity characteristics of COVID-19 patients, in comparison to non-severe COVID-19 patients, those with severe diseases were generally significantly older, and more likely to have underlying diseases such as hypertension, diabetes, and cardiovascular diseases. Of all the studies, only Qu *et al.* reported higher PLR values on admission in non-severe patients compared to severe patients (non-severe: 242.75 ± 173.74 vs. severe: 160.02 ± 51.99; *p* = 0.414) while the remainder demonstrated higher on-admission PLR values in severe compared to non-severe COVID-19 patients.

Two studies performed a receiver operator curve (ROC) analysis to determine the most optimal cut-off level for PLR. Yang *et al.* reported the optimal cut-off level for PLR was 180 [Area under the ROC Curve (AUC) 0.784 (95%CI 0.666–0.901); sensitivity 77%; specificity 44%]^11^ while Sun *et al.* reported the optimal cut-off level was 226.67 [AUC 0.746 (95%CI 0.637–0.854); sensitivity 59.26% (95%CI 38.8%–77.6%); specificity 80.90% (95%CI 71.2%–88.5%)].^16^

### Platelet-to-lymphocyte ratio (PLR) and severity of COVID-19

**Figure 2.**
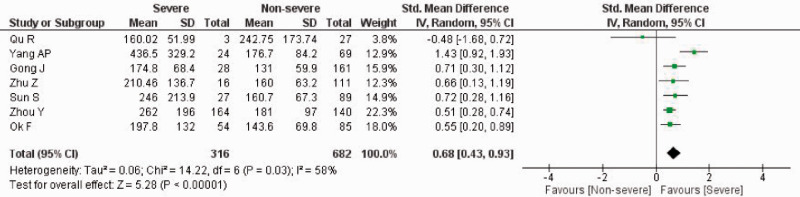
PLR value on admission and severity of COVID-19. Forest Plot using the inverse variance random-effects model showing the association between PLR value on admission and severity of COVID-19 for all included studies.

A pooled effect size meta-analysis was conducted using the random-effects model (n = 998; severe = 316, non-severe = 682) (Figure 2). Overall, patients with severe COVID-19 had a higher PLR value on admission compared to non-severe COVID-19 (SMD 0.68; 95%CI 0.43–0.93). There was significant heterogeneity among the studies (I^2^ = 58%, *p* = 0.03). The funnel plot was visually asymmetrical and indicated a potential risk of publication bias (Figure S1).

Sensitivity analysis by sequentially removing one study at a time did not significantly change the heterogeneity among the studies and the overall pooled effect size. However, the exclusion of the study by Yang *et al.* resulted in no significant heterogeneity between the studies (I^2^ = 0%, *p* = 0.52) (Figure S2). The pooled effect size meta-analysis using the fixed-effects model showed a significantly higher PLR value on admission in severe COVID-19 patients than non-severe COVID-19 patients (SMD 0.57; 95%CI 0.41–0.72).

## Discussion

As of 26 July 2020, COVID-19 has infected approximately 15 million people worldwide, 600 thousand of whom died.^2^ More concerning, those with severe COVID-19 rapidly deteriorate to critical cases, which involve multiorgan failure, leading to death.^19^ Thus, there is an urgent need for healthcare providers to develop readily available biological markers to predict the severity and mortality of COVID-19 at the early stage of the disease to provide the most optimal management.

PLR was initially suggested as an excellent candidate marker for determining the severity and mortality of COVID-19. First, PLR is an established marker of inflammation.^20^ Inflammation plays a considerable role in the pathophysiology of COVID-19, with cytokine storm as a hallmark condition in severe disease and poorer prognosis.^21^ Thus, elevated PLR value suggests an overactive inflammatory response and subsequently, worse prognosis. Second, PLR is sensitive to natural and acquired immune response.^22^ Third, PLR is an inexpensive and readily available measurement that can be used in resource-limited settings. Therefore, our systematic review aims to review the validity of the PLR level on admission as a prognostic indicator in COVID-19 patients.

Our meta-analysis, which included a total of 998 COVID-19 patients, showed that high PLR value was associated with severe COVID-19. Six out of the seven included studies demonstrated similar results with increased PLR on admission found in severe cases of COVID-19 compared to those with mild or moderate diseases. This suggests that elevated PLR on admission among severe COVID-19 patients reflects a higher degree of the cytokine storm. This evidence can be useful for providing specialized treatment to patients with severe COVID-19, as they might require more prolonged hospital admissions.

Although PLR on admission was generally shown to be increased in severe vs. non-severe COVID-19, Qu *et al.* reported a reduced PLR on admission in severe compared to non-severe COVID-19 despite no statistical significance. Inconsistencies between the results of different studies could be problematic, especially for daily clinical applications. However, we noted the rather small sample size of the study. Only three severe cases of COVID-19 were included in the analysis compared to 27 non-severe cases, which could lead to this contradictory result.^10^ Furthermore, the follow-up analysis of PLR in the study showed elevated levels in severe COVID-19 cases upon hospitalization.

To date, there is no universal laboratory reference value for PLR, especially for COVID-19 patients. Of all the included studies, only two studies attempted to determine the optimal cut-off PLR value. Yang *et al.* reported the optimal cut-off PLR value as 180 with AUC of 0.784, specificity of 44%, and sensitivity of 77%.^11^ Meanwhile, Sun *et al.* suggested a cut-off PLR value of 226.67 with AUC of 0.746, specificity of 80.90%, and sensitivity of 59.26%.^16^ This discrepancy warrants the need for further research to determine the most appropriate PLR cut-off value in determining the severity of COVID-19 patients.

As PLR involves a comparison between the absolute platelet and absolute lymphocyte count, a comprehensive understanding of the role of platelets and lymphocytes in COVID-19 is important. Previous meta-analyses have reported that thrombocytopenia and lymphopenia were both associated with severe COVID-19 cases.^23–25^ However, until the writing of this manuscript, the underlying mechanism by which high PLR levels were reported in severe COVID-19 cases remains unclear. A plausible explanation could be that the decrease in absolute lymphocyte counts was much more significant than the decrease in platelet counts, thus increasing the PLR value. Three hypotheses may explain the underlying decrease in platelet count in COVID-19 patients. First, the cytokine storm triggered by SARS-CoV-2 can decrease the synthesis of platelets by destroying bone marrow progenitor cells. SARS-CoV-2 is also postulated to directly affect the production of platelets in the bone marrow. Second, SARS-CoV-2 can induce the generation of autoantibody and immune complex, which may trigger the destruction of platelets. Third, platelets activated during lung injury could be aggregated and be overactively involved in microthrombus formation.^26^ On the other hand, the underlying mechanism for the decreased absolute lymphocyte count is that SARS-CoV-2 triggers pyroptosis in lymphocytes through the activation of NLRP3 inflammasome.^26^ Furthermore, another hypothesis points out the role of the proinflammatory cytokine IL-6 that utilizes the lymphocytes; hence, decreased lymphocyte counts are associated with poor prognosis in COVID-19 patients.

We also observed a significant heterogeneity among the included studies in this meta-analysis. The possible reasons for the high heterogeneity could be due to the distinct interstudy baseline characteristics of the subjects, different number, and proportion of patients with comorbidities as well as the proportion of severe and non-severe cases. Fesih, Qu, and Sun excluded patients with other comorbidities, such as chronic lung diseases, hematological diseases, and liver diseases^10,16,18^ while Gong did not provide any data on the comorbidities of the patients.^14^ Another reason could be due to the small number of included studies in this meta-analysis, which could reduce the accuracy of the heterogeneity analysis.^27^ We performed a sensitivity analysis by sequentially omitting one study at a time and determined that Yang *et al.* was the source of heterogeneity.^11^ However, with the removal of Yang *et al.*, the pooled analysis still showed a statistically significant higher PLR value on admission in severe COVID-19 in comparison to non-severe COVID-19 patients.

This meta-analysis is not without limitations. We acknowledge that only including articles written and published in English would disregard those written in other languages and present with a geographical bias. Moreover, most of the included studies were from China, whereas the majority of confirmed cases and deaths were located in the USA and Europe. The variability in PLR values between different populations could limit the relevance of this finding. Based on the funnel plot, we also identified a potential risk of publication bias. In addition, the limited data presented by the included studies did not allow further stratification of the severe group into severe and critically ill patients. Therefore, further research still needs to be conducted to determine an optimal cut-off value for PLR value to predict severity in COVID-19.

## Conclusion

Our meta-analysis showed that PLR could be used as a novel, cost-effective, and readily available biomarker in determining the severity of COVID-19 patients. Our finding implies that elevated PLR levels on admission can be utilized as a prognostic indicator of severity in COVID-19 patients, especially in resource-limited settings, where there is an urgent need to effectively allocate medical resources and divert attention to patients with poorer prognosis. However, further studies are needed to determine the cut-off value of PLR with the most optimal sensitivity and specificity prior to adaptation in clinical practice.

## Supplemental Material

sj-pdf-1-inc-10.1177_1751143720969587 - Supplemental material for Platelet-to-lymphocyte ratio, a novel biomarker to predict the severity of COVID-19 patients: A systematic review and meta-analysis Click here for additional data file. Supplemental material, sj-pdf-1-inc-10.1177_1751143720969587 for Platelet-to-lymphocyte ratio, a novel biomarker to predict the severity of COVID-19 patients: A systematic review and meta-analysis by Daniel Martin Simadibrata, Bashar Adi Wahyu Pandhita, Muammar Emir Ananta and Tamara Tango in Journal of the Intensive Care Society

## References

[1] Rothan HA Byrareddy SN. The epidemiology and pathogenesis of coronavirus disease (COVID-19) outbreak. J Autoimmun 2020; 109: 102433. 32113704 10.1016/j.jaut.2020.102433 PMC7127067

[2] World Health Organization. *Coronavirus disease (COVID-19): situation report*. Vol. 188. Geneva: World Health Organization, 2020.

[3] Long B Brady WJ Koyfman A, et al. Cardiovascular complications in COVID-19. Am J Emerg Med 2020; 38: 1504–1507. 32317203 10.1016/j.ajem.2020.04.048 PMC7165109

[4] Joob B Wiwanitkit V. COVID-19 can present with a rash and be mistaken for dengue. J Am Acad Dermatol 2020; 82: e177. 32213305 10.1016/j.jaad.2020.03.036 PMC7156802

[5] Wu P Duan F Luo C, et al. Characteristics of ocular findings of patients with coronavirus disease 2019 (COVID-19) in Hubei province, China. JAMA Ophthalmol 2020; 138: 575–578. 32232433 10.1001/jamaophthalmol.2020.1291 PMC7110919

[6] Pennica A Conforti G Falangone F, et al. Clinical management of adult coronavirus infection disease 2019 (COVID-19) positive in the setting of low and medium intensity of care: a short practical review. SN Compr Clin Med 2020; 1–6. 10.1007/s42399-020-00333-w PMC7258606 32838135

[7] Yuki K Fujiogi M Koutsogiannaki S. COVID-19 pathophysiology: a review. Clin Immunol 2020; 215: 108427. 32325252 10.1016/j.clim.2020.108427 PMC7169933

[8] Bonow RO Fonarow GC O’Gara PT, et al. Association of coronavirus disease 2019 (COVID-19) with myocardial injury and mortality. JAMA Cardiol 2020; 5: 751. 32219362 10.1001/jamacardio.2020.1105

[9] Gasparyan AY Ayvazyan L Mukanova U, et al. The platelet-to-lymphocyte ratio as an inflammatory marker in rheumatic diseases. Ann Lab Med 2019; 39: 345–357. 30809980 10.3343/alm.2019.39.4.345 PMC6400713

[10] Qu R Ling Y Zhang Y-H-Z, et al. Platelet-to-lymphocyte ratio is associated with prognosis in patients with coronavirus disease-19. J Med Virol 2020; 92: 1533–1541. 32181903 10.1002/jmv.25767 PMC7228291

[11] Yang A-P Liu J-P Tao W-Q, et al. The diagnostic and predictive role of NLR, d-NLR and PLR in COVID-19 patients. Int Immunopharmacol 2020; 84: 106504. 32304994 10.1016/j.intimp.2020.106504 PMC7152924

[12] World Health Organization. *Report of the WHO-China joint mission on coronavirus disease 2019 (COVID-19)*. GenevaL World Health Organization, 2020.

[13] Hozo SP Djulbegovic B Hozo I. Estimating the mean and variance from the median, range, and the size of a sample. BMC Med Res Methodol 2005; 5: 13. 15840177 10.1186/1471-2288-5-13 PMC1097734

[14] Gong J Ou J Qiu X, et al. A tool for early prediction of severe coronavirus disease 2019 (COVID-19): a multicenter study using the risk nomogram in Wuhan and Guangdong, China. Clin Infect Dis 2020. 10.1093/cid/ciaa443 PMC7184338 32296824

[15] Zhu Z Cai T Fan L, et al. Clinical value of immune-inflammatory parameters to assess the severity of coronavirus disease 2019. Int J Infect Dis 2020; 95: 332–339. 32334118 10.1016/j.ijid.2020.04.041 PMC7195003

[16] Sun S Cai X Wang H, et al. Abnormalities of peripheral blood system in patients with COVID-19 in Wenzhou, China. Clin Chim Acta 2020; 507: 174–180. 32339487 10.1016/j.cca.2020.04.024 PMC7194694

[17] Zhou Y Guo S He Y, et al. COVID-19 is distinct from SARS-CoV-2-negative community-acquired pneumonia. Front Cell Infect Microbiol 2020; 10 : 322. 10.3389/fcimb.2020.00322 PMC7309449 32612961

[18] Ok F Erdogan O Durmus E, et al. Predictive values of blood urea nitrogen/creatinine ratio and other routine blood parameters on disease severity and survival of COVID-19 patients. J Med Virol 2020. 10.1002/jmv.26300 PMC7405288 32662893

[19] Robba C Battaglini D Pelosi P, et al. Multiple organ dysfunction in SARS-CoV-2: MODS-CoV-2. Expert Rev Respir Med 2020; 1–4. 10.1080/17476348.2020.1778470 PMC7441756 32567404

[20] Akboga MK Canpolat U Yuksel M, et al. Platelet to lymphocyte ratio as a novel indicator of inflammation is correlated with the severity of metabolic syndrome: a single center large-scale study. Platelets 2016; 27: 178–183. 26196312 10.3109/09537104.2015.1064518

[21] Merad M Martin JC. Pathological inflammation in patients with COVID-19: a key role for monocytes and macrophages. Nat Rev Immunol 2020; 20: 355–362. 32376901 10.1038/s41577-020-0331-4 PMC7201395

[22] Kartal O Kartal AT. Value of neutrophil to lymphocyte and platelet to lymphocyte ratios in pneumonia. Bratisl Lek Listy 2017; 118: 513–516. 29061056 10.4149/BLL_2017_099

[23] Huang I Pranata R. Lymphopenia in severe coronavirus disease-2019 (COVID-19): systematic review and Meta-analysis. J Intensive Care 2020; 8: 36. 32483488 10.1186/s40560-020-00453-4 PMC7245646

[24] Lippi G Plebani M Henry BM. Thrombocytopenia is associated with severe coronavirus disease 2019 (COVID-19) infections: a meta-analysis. Clin Chim Acta 2020; 506: 145–148. 32178975 10.1016/j.cca.2020.03.022 PMC7102663

[25] Brandon Michael H Maria Helena Santos de O Stefanie B, et al. Hematologic, biochemical and immune biomarker abnormalities associated with severe illness and mortality in coronavirus disease 2019 (COVID-19): a meta-analysis. Clin Chem Lab Med 2020; 58: 1021–1028. 32286245 10.1515/cclm-2020-0369

[26] Yang M. Cell pyroptosis, a potential pathogenic mechanism of 2019-nCoV infection. 2020, 10.2139/ssrn.3527420.

[27] von Hippel PT. The heterogeneity statistic I(2) can be biased in small meta-analyses. BMC Med Res Methodol 2015; 15: 35. 25880989 10.1186/s12874-015-0024-z PMC4410499

