# Supplementary material for: Platelet-to-lymphocyte ratio, a novel biomarker to predict the severity of COVID-19 patients: A systematic review and meta-analysis
Source: J Intensive Care Soc. 2020 Nov 2;23(1):20–6. doi: 10.1177/1751143720969587 (PMC7649648; doi:10.1177/1751143720969587)

List of Supplementary Files

Table S1. PRISMA checklist

Table S2. Search strategy for peer-reviewed electronic databases

Table S3. Risk of bias (quality) assessment of the included literature with Newcastle Ottawa Scale (NOS)

Figure S1. Publication bias of studies included in the meta-analysis

Figure S2. Meta-analysis of all included studies (without Yang AP et al)

Table S1. PRISMA checklist

Section/topic	#	Checklist item	Reported on page #
**TITLE**			
Title	1	Identify the report as a systematic review, meta-analysis, or both.	1
**ABSTRACT**			
Structured summary	2	Provide a structured summary including, as applicable: background; objectives; data sources; study eligibility criteria, participants, and interventions; study appraisal and synthesis methods; results; limitations; conclusions and implications of key findings; systematic review registration number.	2, 3
**INTRODUCTION**			
Rationale	3	Describe the rationale for the review in the context of what is already known.	4
Objectives	4	Provide an explicit statement of questions being addressed with reference to participants, interventions, comparisons, outcomes, and study design (PICOS).	4, 5
**METHODS**			
Protocol and registration	5	Indicate if a review protocol exists, if and where it can be accessed (e.g., Web address), and, if available, provide registration information including registration number.	6
Eligibility criteria	6	Specify study characteristics (e.g., PICOS, length of follow-up) and report characteristics (e.g., years considered, language, publication status) used as criteria for eligibility, giving rationale.	6
Information sources	7	Describe all information sources (e.g., databases with dates of coverage, contact with study authors to identify additional studies) in the search and date last searched.	6
Search	8	Present full electronic search strategy for at least one database, including any limits used, such that it could be repeated.	6, 7, Supplementary Material
Study selection	9	State the process for selecting studies (i.e., screening, eligibility, included in systematic review, and, if applicable, included in the meta-analysis).	7
Data collection process	10	Describe method of data extraction from reports (e.g., piloted forms, independently, in duplicate) and any processes for obtaining and confirming data from investigators.	7
Data items	11	List and define all variables for which data were sought (e.g., PICOS, funding sources) and any assumptions and simplifications made.	7
Risk of bias in individual studies	12	Describe methods used for assessing risk of bias of individual studies (including specification of whether this was done at the study or outcome level), and how this information is to be used in any data synthesis.	7
Summary measures	13	State the principal summary measures (e.g., risk ratio, difference in means).	7, 8
Synthesis of results	14	Describe the methods of handling data and combining results of studies, if done, including measures of consistency (e.g., I^2) for each meta-analysis.	7, 8

Section/topic	#	Checklist item	Reported on page #
Risk of bias across studies	15	Specify any assessment of risk of bias that may affect the cumulative evidence (e.g., publication bias, selective reporting within studies).	8
Additional analyses	16	Describe methods of additional analyses (e.g., sensitivity or subgroup analyses, meta-regression), if done, indicating which were pre-specified.	8
**RESULTS**			
Study selection	17	Give numbers of studies screened, assessed for eligibility, and included in the review, with reasons for exclusions at each stage, ideally with a flow diagram.	9, 21 supplementary material
Study characteristics	18	For each study, present characteristics for which data were extracted (e.g., study size, PICOS, follow-up period) and provide the citations.	9, 20, supplementary material
Risk of bias within studies	19	Present data on risk of bias of each study and, if available, any outcome level assessment (see item 12).	9, supplementary material
Results of individual studies	20	For all outcomes considered (benefits or harms), present, for each study: (a) simple summary data for each intervention group (b) effect estimates and confidence intervals, ideally with a forest plot.	9, 10, 20
Synthesis of results	21	Present results of each meta-analysis done, including confidence intervals and measures of consistency.	9, 10, 22
Risk of bias across studies	22	Present results of any assessment of risk of bias across studies (see Item 15).	10, supplementary material
Additional analysis	23	Give results of additional analyses, if done (e.g., sensitivity or subgroup analyses, meta-regression [see Item 16]).	10, supplementary material
**DISCUSSION**			
Summary of evidence	24	Summarize the main findings including the strength of evidence for each main outcome; consider their relevance to key groups (e.g., healthcare providers, users, and policy makers).	11-13
Limitations	25	Discuss limitations at study and outcome level (e.g., risk of bias), and at review-level (e.g., incomplete retrieval of identified research, reporting bias).	13, 14
Conclusions	26	Provide a general interpretation of the results in the context of other evidence, and implications for future research.	15
**FUNDING**			
Funding	27	Describe sources of funding for the systematic review and other support (e.g., supply of data); role of funders for the systematic review.	17

From: Moher D, Liberati A, Tetzlaff J, Altman DG, The PRISMA Group (2009). Preferred Reporting Items for Systematic Reviews and Meta-Analyses: The PRISMA Statement. PLoS Med 6(7): e1000097. doi:10.1371/journal.pmed1000097

Table S2. Search strategy for peer-reviewed electronic databases. The results showed the number of papers retrieved as of 23 July 2020.

Database	Search Terms	Hits
**Ovid MEDLINE**	1. COVID-19<br>2. COVID19<br>3. COVID<br>4. “Coronavirus disease 2019”<br>5. 2019-nCoV<br>6. SARS-CoV-2<br>7. 1 OR 2 OR 3 OR 4 OR 5 OR 6<br>8. platelet-to-lymphocyte<br>9. PLR<br>10. 8 OR 9<br>11. Sever*<br>12. “Intensive care unit”<br>13. ICU<br>14. Mortality<br>15. Death<br>16. Non-survivor<br>17. 11 OR 12 OR 13 OR 14 OR 15 OR 16<br>18. 7 AND 10 AND 17	12
**EMBASE**	1. COVID-19<br>2. COVID19<br>3. COVID<br>4. “Coronavirus disease 2019”<br>5. 2019-nCoV<br>6. SARS-CoV-2<br>7. 1 OR 2 OR 3 OR 4 OR 5 OR 6<br>8. platelet-to-lymphocyte<br>9. PLR<br>10. 8 OR 9<br>11. Sever*<br>12. “Intensive care unit”<br>13. ICU<br>14. Mortality<br>15. Death<br>16. Non-survivor<br>17. 11 OR 12 OR 13 OR 14 OR 15 OR 16<br>18. 7 AND 10 AND 16	5
**SCOPUS**	(TITLE-ABS-KEY(COVID-19) OR TITLE-ABS-KEY(COVID19) OR TITLE-ABS-KEY(COVID) OR TITLE-ABS-KEY("coronavirus disease 2019") OR TITLE-ABS-KEY(2019-ncov) OR TITLE-ABS-KEY(sars-cov-2)) AND (TITLE-ABS-KEY(platelet-to-lymphocyte) OR TITLE-ABS-KEY(PLR)) AND (TITLE-ABS-KEY(sever*) OR TITLE-ABS-KEY(“intensive care unit”) OR TITLE-ABS-KEY(ICU) OR TITLE-ABS-KEY(mortality) OR TITLE-ABS-KEY(death) OR TITLE-ABS-KEY(non-survivor))	6
**The Cochrane Library**	("Covid-19" OR "COVID19" OR "COVID" OR "coronavirus disease 2019" OR "2019-nCoV" OR "SARS-CoV-2") in Title Abstract Keyword AND (platelet-to-lymphocyte OR PLR) in Title Abstract Keyword AND (sever* OR "intensive care unit" OR ICU OR mortality OR death OR non-survivor)	0
**Total**		23

Table S3. Risk of bias (quality) assessment of the included literature with the Newcastle Ottawa Scale (NOS)

Author	Date of Publication	Study Location	Publication Type	Study Period	Study Design	Selection	Comparability	Outcome/Exposure	Risk of bias score
Qu R et al	12/03/2020	Huizhou, China	Peer-reviewed	Jan 20 to 21 Feb 20	Retrospective Observational	**	*	***	6
Yang AP et al	13/04/2020	China	Peer-reviewed	NR	Retrospective Observational	**	**	***	7
Gong J et al	16/04/2020	Wuhan and Guangzhou, China	Peer-reviewed	20 Jan 20 to 02 Mar 20	Retrospective Observational	**	**	***	7
Zhu Z et al	17/04/2020	Ningbo, Zhejiang, China	Peer-reviewed	23 Jan 20 to 20 Feb 20	Retrospective Observational	**	**	***	7
Sun S et al	24/04/2020	Wenzhou, China	Peer-reviewed	19 Jan 20 to 20 Feb 20	Retrospective Observational	**	*	***	6
Zhou Y et al	16/06/2020	Wuhan, China	Peer-reviewed	1 Feb 20 to 15 Mar 20	Retrospective Observational	**	*	***	6
Ok F et al	10/07/2020	Siirt, Turkey	Peer-reviewed	Apr 20 to May 20	Retrospective Observational	**	**	***	7

Figure S1. Publication bias of studies included in the meta-analysis. Funnel Plot representing all the included studies. SMD = Standardized Mean Difference, SE(SMD) = Standard Error of the SMD.

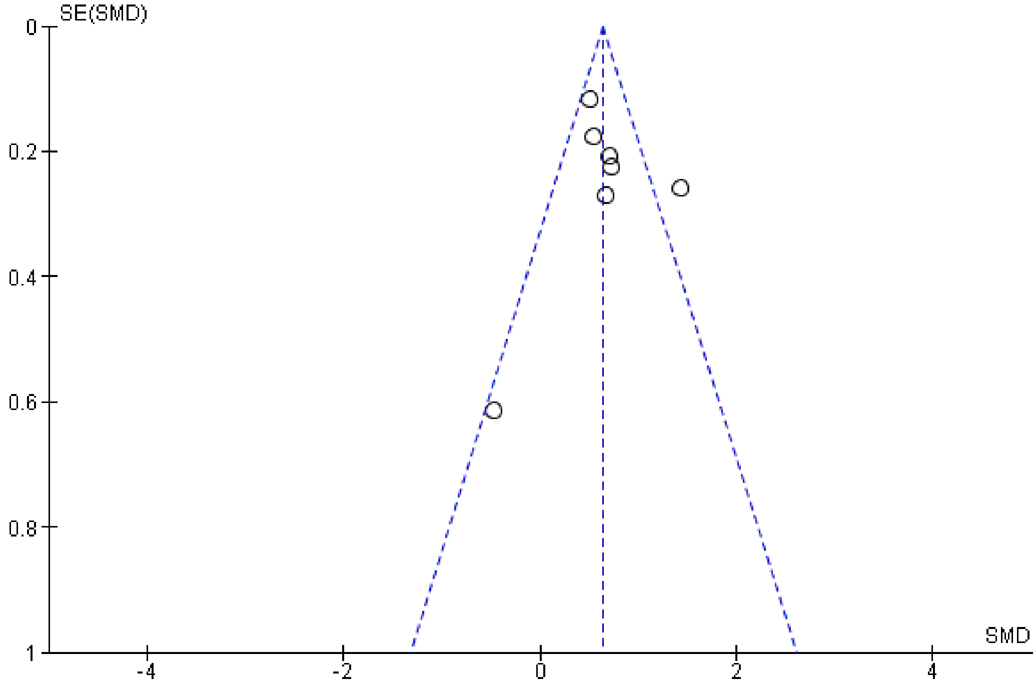

Figure S2. Meta-analysis of all included studies (without Yang AP et al). Forest Plot using the inverse variance fixed-effect model showing the association between PLR value on admission and severity of COVID-19

	Severe			Non-severe				Std. Mean Difference
Study or Subgroup	Mean	SD	Total	Mean	SD	Total	Weight	IV, Fixed, 95% CI
Qu R	160.02	51.99	3	242.75	173.74	27	1.6%	-0.48 [-1.68, 0.72]
Gong J	174.8	68.4	28	131	59.9	161	14.1%	0.71 [0.30, 1.12]
Zhu Z	210.46	136.7	16	160	63.2	111	8.3%	0.66 [0.13, 1.19]
Sun S	246	213.9	27	160.7	67.3	89	12.0%	0.72 [0.28, 1.16]
Zhou Y	262	196	164	181	97	140	44.5%	0.51 [0.28, 0.74]
Ok F	197.8	132	54	143.6	69.8	85	19.4%	0.55 [0.20, 0.89]
**Total (95% CI)**			**292**			**613**	**100.0%**	**0.57 [0.41, 0.72]**

Heterogeneity: Chi² = 4.23, df = 5 (P = 0.52); I² = 0%
Test for overall effect: Z = 7.27 (P < 0.00001)

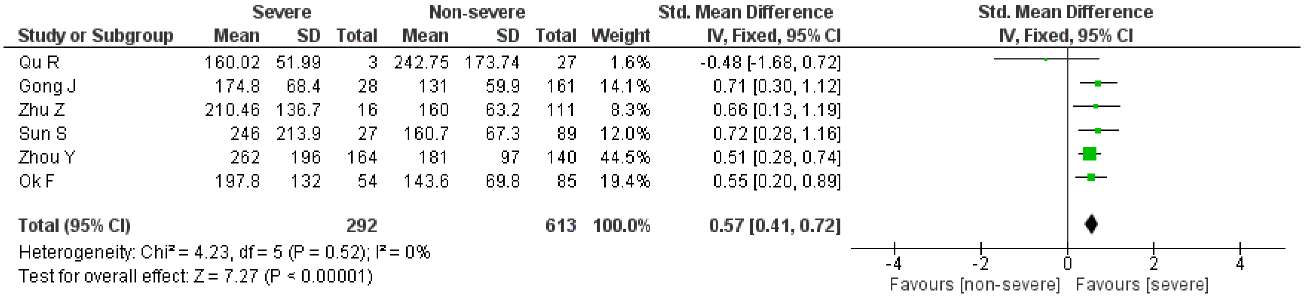

Supplement: sj-pdf-1-inc-10.1177_1751143720969587 - Supplemental material for Platelet-to-lymphocyte ratio, a novel biomarker to predict the severity of COVID-19 patients: A systematic review and meta-analysis [file sj-pdf-1-inc-10.1177_1751143720969587.pdf]
